# Supplementary material for: Structural basis of mitochondrial membrane bending by the I–II–III2–IV2 supercomplex
Source: Nature. 2023 Mar 22;615(7954):934–8. doi: 10.1038/s41586-023-05817-y (PMC10060162; doi:10.1038/s41586-023-05817-y)
Supplement: Supplementary file 1 — This file contains Supplementary Table 1: Data collection and model statistics; Supplementary Table 2: List of proteins and comments; and Source Data for Extended Data Fig. 2. [file 41586_2023_5817_MOESM1_ESM.pdf]

nature portfolio

https://doi.org/10.1038/s41586-023-05817-y

Supplementary information

Structural basis of mitochondrial membrane bending by the I–II–III_2–IV_2 supercomplex

In the format provided by the authors and unedited

Structural basis of mitochondrial membrane bending by I-II-III_2-IV_2 supercomplex

Alexander Mühleip[1†], Rasmus Kock Flygaard[1,2†], Rozbeh Baradaran[1,3], Outi Haapanen[4], Thomas Gruhl[5], Victor Tobiasson[1], Amandine Maréchal[5,6], Vivek Sharma[4,7], Alexey Amunts[1*]

[1] Science for Life Laboratory, Department of Biochemistry and Biophysics, Stockholm University, 17165 Solna, Sweden.

[2] Current address: Department of Molecular Biology and Genetics, Danish Research Institute of Translational Neuroscience - DANDRITE, Nordic EMBL Partnership for Molecular Medicine, Aarhus University, 8000 Aarhus C, Denmark.

[3] Current address: MRC Laboratory of Molecular Biology, Cambridge, United Kingdom.

[4] Department of Physics, University of Helsinki, 00014 Helsinki, Finland.

[5] Institute of Structural and Molecular Biology, Birkbeck College, London, WC1E 7HX, UK.

[6] Institute of Structural and Molecular Biology, University College London, London, WC1E 6BT, UK.

[7] HiLIFE Institute of Biotechnology, University of Helsinki, 00014 Helsinki, Finland.

† These authors contributed equally to this work.

* Correspondence to: amunts@scilifelab.se

SUPPLEMENTARY INFORMATION

Table of contents:

SI Table 1. Data collection and model statistics

SI Table 2. List of proteins and comments

Supplementary Video 1. Coarse-grained molecular dynamics simulation of the *T. thermophila* supercomplex. First 800 ns of the MD simulation starting from an initially planar membrane reveals a deformation of the bilayer into a curved topology to accommodate the membrane protein complex.

Supplementary Video 2. Coarse-grained molecular dynamics simulation of pure lipid bilayer. Membrane shows fluctuations, however, a stable curved architecture (as in the case of supercomplex) is not observed.

Supplementary Video 3. Annular lipid shell of the *T. thermophila* supercomplex. Final frame of the coarse-grained MD-simulation with supercomplex and surrounding annular lipids shown, highlighting the curved shape of the lipid belt.

Source data for Extended Data Figure 2.

Supplementary Information Table 1. Data collection and model statistics

Data collection	CI	CII	$CIII_2$	CIV_2	CI-II-III_2-IV_2
Microscope	Titan Krios				
Voltage (kV)	300				
Camera	K2 Summit				
Magnification	165,000				
Exposure ($e^-/Å^2$)	30.9				
Pixel size (Å)	0.83				
Defocus range (μm)	0.6-2.6				
Movies collected	26,063				
Frames / movie	20				
Data processing					
Initial particles	1,664,103				
Final particles	138,746				
Symmetry	C1	C1	C1	C1	C1
Map resolution (Å)	2.9	2.8	2.8	2.6	2.9
- FSC threshold	0.143	0.143	0.143	0.143	0.143
B-factor sharpening	46.8	49.1	52.2	45.4	49.9
EMDB ID	EMD-	EMD-	EMD-	EMD-	EMD-
Model refinement statistics					
CC (map/model)	0.82	0.82	0.81	0.84	0.79
Resolution (map/model)	2.99	3.3	3.18	2.89	3.36
- FSC threshold	0.5	0.5	0.5	0.5	0.5
Atoms (not H)	234277	41399	94264	436066	806006
Residues	13712	2409	5438	23792	45351
Lipids/CoQ	59/0	11/1	32/3	214/8	316/12
Model B-factor ($Å^2$)					
- Protein	53.2	100.46	65.66	52.8	91.23
- Ligands	63.83	107.88	78.66	72.16	111.10
Rotamer outliers (%)	0.51%	0.62%	0.53%	1.18%	0.76
Ramachandran (%)					
- Outliers	0.01%	0.21%	0.00%	0.01%	0.02%
- Allowed	2.07%	3.28%	2.43%	1.75%	1.77
- Favoured	97.92%	96.51%	97.57%	98.25%	98.22
Clash score	4.12	3.91	5.68	4.22	4.63
MolProbity score	1.21	1.41	1.40	1.26	1.24
RMSD					
- Bonds (Å)	0.002	0.004	0.002	0.002	0.002
- Angles (°)	0.407	0.459	0.447	0.503	0.562
PDB ID					

Supplementary Information Table 2. List of proteins and comments

Subunit name	Gene name	UniProt ID	Encoded	Residues in total	Comments
**Complex-I:**					
ND1a	nad1_a	Q950Y3	mt	284	Corresponds to NuoH, Nqo8
ND1b	nad1_b	NP_149380	mt	59	Mt-ND1 C-terminal extension, separate gene
ND2a	ymf65	Q951A3	mt	360	Corresponds to NuoN, Nqo14
ND2b	nad2	Q951B2	mt	178	Mt-ND2 C-terminal extension, separate gene
Mt-ND3	nad3	Q950Z7	mt	121	Corresponds to NuoA, Nqo7
ND4	nad4	Q950X9	mt	505	Corresponds to NuoM, Nqo13
MT-ND4L	ymf58	Q950Z5	mt	116	Corresponds to NuoK, Nqo11
ND5a	nad5	Q950Z0	mt	750	Corresponds to NuoL, Nqo12
ND5b	ymf57	Q951C2	mt	100	Mt-ND5 C-terminal extension, separate gene
ND6	ymf62	Q950Y2	mt	255	Corresponds to NuoJ, Nqo10
NDUV1	TTHERM_00193910	Q23KE4	nuclear	474	Corresponds to NuoF, Nqo1
NDUV2	TTHERM_00335630	I7MEP0	nuclear	274	Corresponds to NuoE, Nqo2

NDUS1	TTHERM_00194 260	Q23KA9	nuclear	718	Corresponds to NuoG, Nqo3
NDUS2	nad7	Q951B1	mt	442	Corresponds to NuoD, Nqo4
NDUS3	nad9_2	Q950Z3	mt	198	Corresponds to NuoC, Nqo5
NDUS4	TTHERM_00526 930	I7MK61	nuclear	185	Corresponds to AQDQ subunit
NDUS5	TTHERM_00012 9647	W7X4R4	nuclear	94	
NDUS6	TTHERM_00497 570	I7MK02	nuclear	132	Zinc-finger protein, contains a Zn^{2+} ion
NDUS7	nad10	Q951B4	mt	162	Corresponds to NuoB, Nqo6
NDUS8	TTHERM_00294 640	I7MDW5	nuclear	236	Corresponds to NuoI, Nqo9
NDUA1	TTHERM_00455 560	I7MI60	nuclear	94	
NDUA2	TTHERM_00659 070	I7MA77	nuclear	103	
NDUA3	TTHERM_00148 710	I7M9B3	nuclear	135	
NDUA5	TTHERM_01161 000	Q23ND5	nuclear	206	
NDUA6	TTHERM_00933 070	I7M2Y3	nuclear	172	
NDUA7	TTHERM_00399 360	I7MIJ7	nuclear	282	
NDUA8	TTHERM_00464 930	I7MMF4	nuclear	238	

NDUA9	TTHERM_00557760	I7MLH2	nuclear	362	NAD-dependent epimerase/dehydratase family protein
NDUA11	TTHERM_00945210	Q24F24	nuclear	213	
NDUA12	TTHERM_00194499	A4VDQ6	nuclear	194	
NDUA13	TTHERM_00149260	I7M2U4	nuclear	175	
NDUAB1-α	TTHERM_01005100	Q22XT6	nuclear	138	Different isoform to NDUAB1-β
NDUAB1-β	TTHERM_00470710	I7MD12	nuclear	133	Acyl carrier protein
NDUB3	TTHERM_00446569	A4VD20	nuclear	83	
NDUB4	TTHERM_00310880	I7MG29	nuclear	126	
NDUB6	TTHERM_00430000	Q231G0	nuclear	129	
NDUB7	TTHERM_00402070	I7MIM0	nuclear	120	
NDUB8	TTHERM_00481330	I7M855	nuclear	207	
NDUB9	TTHERM_00985010	Q233X7	nuclear	189	
NDUB10	TTHERM_00193750	Q23KG0	nuclear	188	
NDUB11	TTHERM_00114380	Q22Z32	nuclear	214	
NDUB15	TTHERM_000614679	-	nuclear	147	Wrong gene annotation – change translation ORF to 5′-3′ in frame 3

NDUC2	TTHERM_00160690	Q22W63	nuclear	102	
NDUCA1	TTHERM_00136440	I7M8Q7	nuclear	346	carbonic anhydrase-like protein (isoform 1)
NDUCA2	TTHERM_01005010	Q22XU5	nuclear	257	carbonic anhydrase-like protein (isoform 2)
NDUCA3	TTHERM_00541460	I7M6S0	nuclear	233	carbonic anhydrase-like protein (isoform 3)
NDUX1	TTHERM_00715850	I7LT42	nuclear	150	
NDUFX	TTHERM_00161210	Q22W11	nuclear	172	2Fe2S cluster-binding protein
NDUTX	TTHERM_01205250	Q22AI5	nuclear	166	Thioredoxin, TRX family
NDUJ1	TTHERM_00938750	Q22DR7	nuclear	317	DnaJ domain protein
NDUPH1	TTHERM_00697370	Q24C39	nuclear	251	
NDUPH2	TTHERM_00193950	Q23KE0	nuclear	189	
NDUTT1	TTHERM_00925340	Q22E24	nuclear	516	lipid A-disaccharide synthase
NDUTT2	TTHERM_01000190	Q24HK5	nuclear	333	Acyl-CoA synthetase (AMP-forming)/AMP-acid ligase II
NDUTT3	TTHERM_00268000	I7LUQ4	nuclear	311	
NDUTT4	TTHERM_00295430	I7MIE0	nuclear	212	
NDUTT5	TTHERM_00649080	I7LT77	nuclear	205	

NDUTT6	TTHERM_00334340	I7M1N8	nuclear	144	
NDUTT7	TTHERM_00637590	Q22HE4	nuclear	143	
NDUTT8	TTHERM_00006120	Q22SC4	nuclear	135	
NDUTT9	TTHERM_00653670	Q23B10	nuclear	136	
NDUTT10	TTHERM_00616320	I7MAF0	nuclear	127	
NDUTT11	TTHERM_00185570	Q22T55	nuclear	113	
NDUTT12	TTHERM_00835330	Q22E95	nuclear	93	
NDUTT13	TTHERM_00992800	Q22DC2	nuclear	73	
NDUTT14	TTHERM_00399460	I7MIK1	nuclear	71	
NDUTT15	TTHERM_000637389	-	nuclear	237	Wrong gene annotation – change translation ORF to 3′-5′ in frame 1 Binds ADP-Mg^{2+}
NDUTT16	TTHERM_001243407	W7XFJ5	nuclear	119	Is required for I-IV_2 interface stability. Stabilises ND5a Nt extension. Wrong gene annotation – change translation ORF to 3′-5′ in frame 3
NDUTT17	TTHERM_000532499	-	nuclear	125	Wrong gene annotation – change translation ORF to 3′-5′ in frame 2

**Complex-II:**					
SDHA	TTHERM_00047080	Q23DI3	nuclear	636	
SDHB	TTHERM_00241700	I7M403	nuclear	312	
SDHC	TTHERM_00387120	Q23RH8	nuclear	60	
SDHD	AF396436.1 (mt-genome sequence)	-	mt	43	Gene lies between ymf66 and ymf76 on mtDNA. Translation 3′-5′ in frame 1
SDHTT1	TTHERM_00571650	Q24I09	nuclear	322	contains heme c
SDHTT2	TTHERM_00532090	Q248F8	nuclear	296	
SDHTT3	TTHERM_00283850	I7MEX7	nuclear	198	
SDHTT4	TTHERM_00658950	I7LX66	nuclear	195	Diphthamide synthesis protein
SDHTT5	TTHERM_00601860	Q22YL0	nuclear	114	
SDHTT6	TTHERM_00626980	Q23S01	nuclear	103	
SDHTT7	TTHERM_00713350	Q24CW6	nuclear	93	
SDHTT8	TTHERM_000287919	W7XBF5	nuclear	89	
SDHTT9	TTHERM_00637670	Q22HD6	nuclear	76	
SDHTT10	TTHERM_001034353	W7XF00	nuclear	62	

SDHTT11	DY684362.1 (EST, mRNA sequence)	-	nuclear	46	Gene translation should be 3′-5′ in frame 1
**Complex-III:**					
MPP-β	TTHERM_00502380	I7MGU2	nuclear	513	
MPP-α	TTHERM_00836690	I7MJ25	nuclear	482	
COB	cob	Q950Z1	mt	426	contains heme bL and bH
CYC1	TTHERM_00918500	Q24IM5	nuclear	319	Contains heme c
UQCRFS1	TTHERM_00295080	I7MIC7	nuclear	269	Contains 2Fe2S cluster
UQCRH	TTHERM_00194690	Q23K66	nuclear	86	cytochrome bc1 hinge proteins
UQCRQ	TTHERM_00765330	I7M484	nuclear	130	
UQCR9	TTHERM_00456790	I7MM45	nuclear	119	
UQCR10	TTHERM_00218930	I7MFL6	nuclear	62	
UQCRTT1	TTHERM_00382330	Q23F81	nuclear	328	
UQCRTT2	EV837551.1 (EST, mRNA sequence)	-	nuclear	41	Gene translation should be 5′-3′ in frame 3
UQCRTT3	-	-	-	66	Unidentified subunit

**Complex-IV:**					
COX1	cox1	Q950Y4	mt	688	
COX2	cox2	Q950Y9	mt	604	
COX3a	ymf67	Q950Y7	mt	453	structurally replaces H1 of COX3
COX3b	ymf68	Q950Y6	mt	594	
COX5B	TTHERM_00378620	Q23FF5	nuclear	637	
COX6A	TTHERM_000723218	W7XCY5	nuclear	130	
COX6B	TTHERM_00568030	Q24I72	nuclear	230	
COX6C	TTHERM_00046170	Q23DS4	nuclear	103	
COX7A	TTHERM_00151250	I7MGF9	nuclear	133	
COX7C	TTHERM_000470561	W7X287	nuclear	236	
COX6BL	TTHERM_00218340	I7LVX0	nuclear	88	
COX17L	TTHERM_01043280	Q22CI1	nuclear	990	CTF/NF-I domain-containing protein
NDUA4	TTHERM_001001528	W7WZP1	nuclear	220	(Named COXTT13 in Zhou et al.)
COXMC1	TTHERM_00127269	A4VDV3	nuclear	346	Oxoglutarate/malate translocator protein

COXMC2	TTHERM_00621600	Q23M99	nuclear	318	2-oxoglutarate/malate carrier protein
COXMC3	TTHERM_00112650	Q22ZA6	nuclear	330	
COXBP	TTHERM_00016360	Q22RF2	nuclear	685	BBC53 chromosome condensation regulator RCC1
COXTIM1	TTHERM_000528460	W7X3D6	nuclear	72	Tim10/DDP family zinc finger proteins
COXTIM2	TTHERM_000703379	W7XDM6	nuclear	72	part of TIM9.10 hexamer
COXTIM3	TTHERM_00433490	Q231A8	nuclear	93	part of TIM9.10 hexamer
COXTIM4	TTHERM_01289060	Q22A35	nuclear	68	part of TIM9.10 hexamer
COXTIM5	TTHERM_00028510	Q22N23	nuclear	81	part of TIM9.10 hexamer
COXTIM6	TTHERM_00805850	Q233U0	nuclear	72	part of TIM9.10 hexamer
COXFS	TTHERM_00133510	I7M8P0	nuclear	188	Fe-binding zinc finger CDGSH type protein
COXAC	TTHERM_00695750	Q24C97	nuclear	127	
Ymf70	ymf70	Q950Y0	mt	89	
Ymf75	ymf75	Q951A7	mt	190	
COXTT1	TTHERM_00361490	Q22PJ5	nuclear	490	
COXTT2	TTHERM_00721790	Q22FX8	nuclear	473	Protein phosphatase 2C

COXTT4	TTHERM_00338280	I7M1Q4	nuclear	402	SURF1 family protein
COXTT5	TTHERM_00046440	Q23DP7	nuclear	385	TraB family protein
COXTT6	TTHERM_00047230	Q23DG8	nuclear	348	
COXTT7	TTHERM_00675650	Q23DZ5	nuclear	318	
COXTT8	TTHERM_00070850	I7LTZ4	nuclear	318	SURF1 family protein
COXTT9	TTHERM_00525160	I7LY65	nuclear	252	
COXTT10	TTHERM_00420130	I7MD70	nuclear	234	
COXTT11	TTHERM_000939159	W7X4J9	nuclear	231	
COXTT12	TTHERM_00455090	I7M3P9	nuclear	215	
COXTT14	TTHERM_00530650	I7LZX8	nuclear	210	
COXTT15	TTHERM_00641250	Q23F08	nuclear	193	
COXTT16	TTHERM_00218840	I7M8Y9	nuclear	175	AAA protein fold
COXTT17	TTHERM_00049040	Q23D87	nuclear	173	
COXTT18	TTHERM_00218570	I7MKT6	nuclear	173	
COXTT19	TTHERM_00433830	Q230X6	nuclear	170	
COXTT20	TTHERM_00794470	Q23VY4	nuclear	158	

COXTT21	TTHERM_00938940	Q22DP8	nuclear	154	
COXTT22	TTHERM_00691100	I7MFV5	nuclear	149	
COXTT23	TTHERM_00666370	Q23TE5	nuclear	124	
COXTT24	TTHERM_00161000	Q22W32	nuclear	122	
COXTT25	TTHERM_00151580	I7M9E7	nuclear	105	
COXTT26	TTHERM_00485790	I7LTF1	nuclear	90	
COXTT27	TTHERM_000302101	W7XDH2	nuclear	212	(Including subunit COXTT3 in Zhou et al.)
COXTT28	TTHERM_000933397	W7X912	nuclear	171	

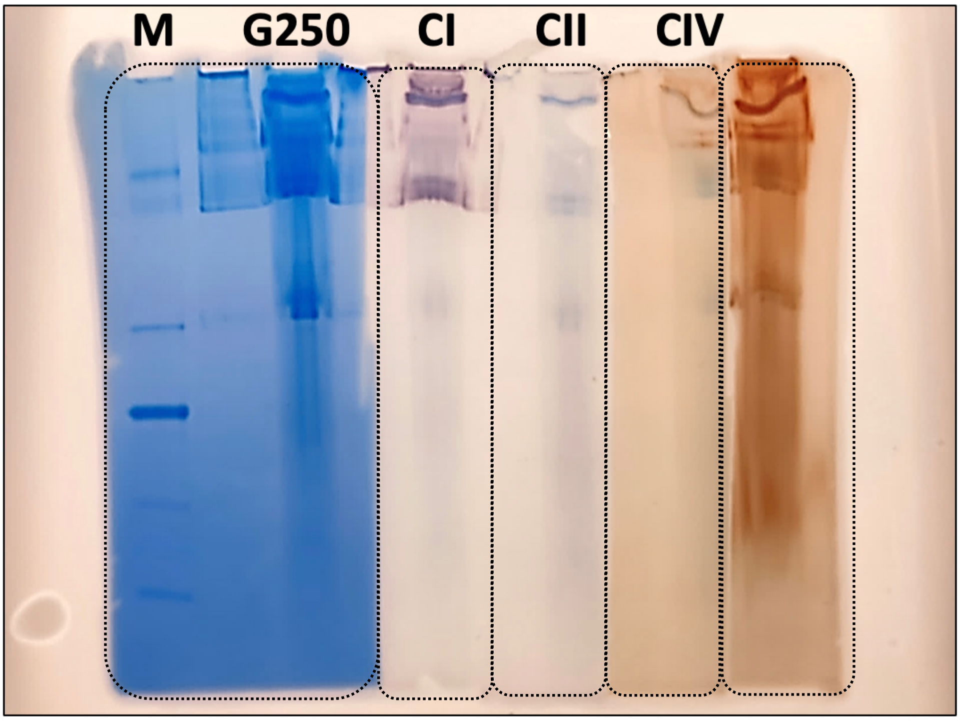

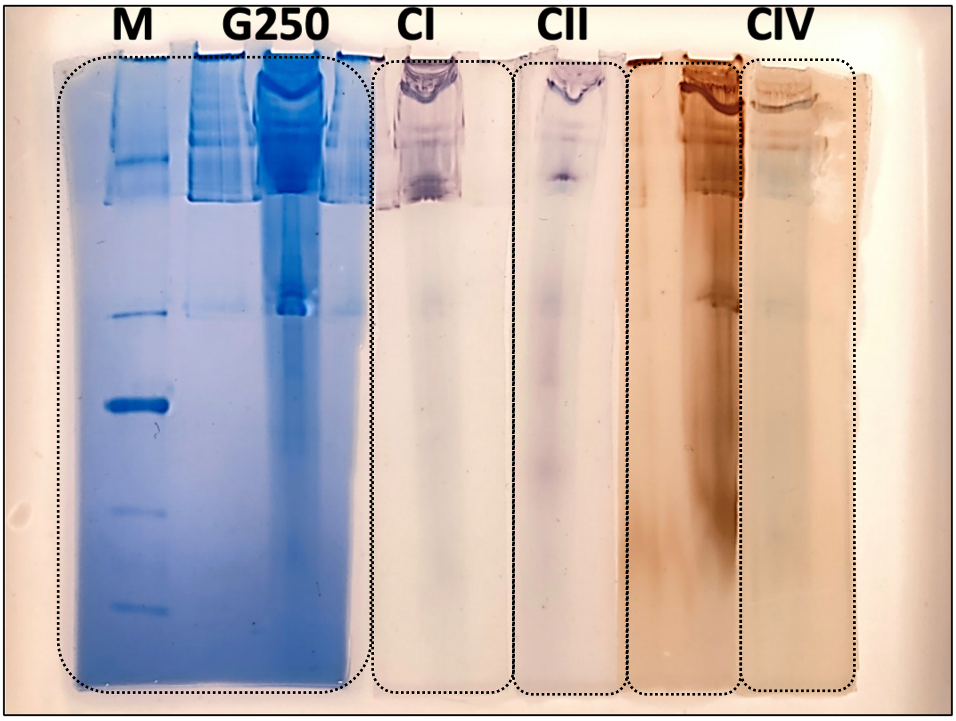

Source data for Extended Data Figure 2. CN-PAGE was performed to separate protein assemblies within the final sucrose cushion sample. After electrophoresis, the gel lanes were cut and incubated with different reagents to reveal the presence of proteins in the marker (M) and sample (G250) lanes, active complex I (CI), complex II (CII) or complex IV (CIV) within remaining sample lanes. The lanes were then juxtaposed to reconstruct the full gel. The two CN-PAGE gels were obtained from two separate supercomplex preparations.
